# Aniridia associated with congenital aphakia and secondary glaucoma

**DOI:** 10.4103/0301-4738.53061

**Published:** 2009

**Authors:** Mayur Moreker, Rajul Parikh, Shefali R Parikh, Ravi Thomas

**Affiliations:** 1 VST Center for Glaucoma Care, L.V. Prasad Eye Institute, Hyderabad, India; 2 Bombay City Eye Institute and Research Center, Mumbai, India; 3 Queensland Eye Institute, Brisbane, Australia; 4 University of Queenslad, Brisbane, Australia

**Keywords:** Aniridia, congenital aphakia, secondary glaucoma

## Abstract

We report a case of aniridia associated with congenital aphakia and secondary glaucoma. A 35-year-old male presented with aniridia, congenital aphakia and secondary glaucoma in both eyes. After an unsuccessful medical management, he underwent trabeculectomy with mitomycin C and anterior vitrectomy under local anesthesia in his left eye. Postoperatively, at the end of six months, intraocular pressure (IOP) in his left eye was controlled without medications. This case highlights the rare association of aniridia with congenital aphakia and secondary glaucoma.

Aniridia is a phenotypically heterogeneous condition that can be inherited as an autosomal dominant disorder or as part of several systemic syndromes. It has been linked to Chromosomes 1 and 2 and associated with the deletion of the p-13 band of Chromosome 11.[1] Aniridia involves not only the iris, but also the retina, optic nerve, lens and cornea.[2] Visual acuity deteriorates as a result of nystagmus, glaucoma, cataract, corneal opacities and retinal hypoplasia. There are numerous reports of association of aniridia with congenital cataract but there is no report in literature showing association of aniridia and congenital aphakia. We report a patient with aniridia, congenital aphakia and secondary glaucoma.

## Case Report

**Figure 1:**
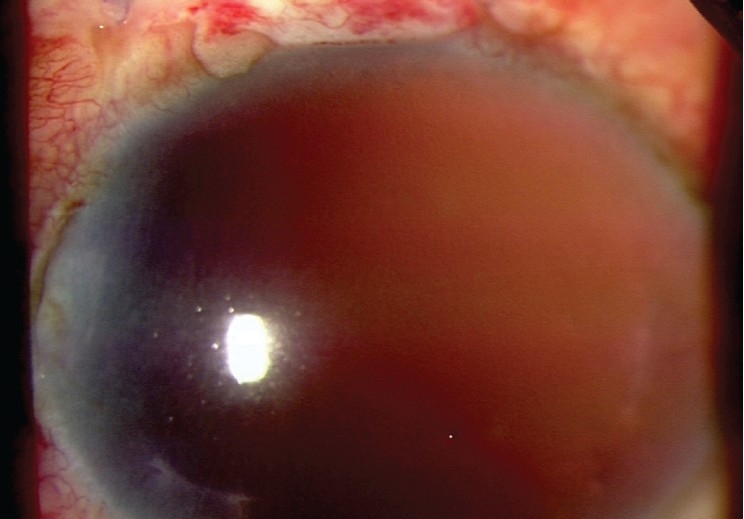
Left eye of the patient with aphakia and aniridia. The patient's left cornea has mild haze. Aniridia and aphakia can be noted

A 35-year-old male presented with complaints of decreased vision in both eyes for 15 years. There was no history of intraocular surgery in either eye. His best-corrected visual acuity was no perception of light in the right eye and 20/400 in the left eye with + 9.0 diopter sphere (Dsph). Horizontal pendular nystagmus was noted in both eyes. Examination of the right eye revealed corneal stromal edema and an intercalary staphyloma. The cornea in the left eye had mild corneal haze. Both eyes had aniridia and were aphakic. The view in the left eye was clearer and showed aphakia with total absence of zonules [Fig. 1]. Intraocular pressure (IOP) measured by Goldmann applanation tonometry was 28 mm Hg and 36 mm Hg in the right and left eye respectively. Corneal edema obscured visualization of the angle in the right eye. Gonioscopy in the left eye with a four-mirror lens showed open angles up to the cilliary body inferiorly; the stump of the iris had formed peripheral anterior synechia (PAS) superiorly. Fundus details were not clear in the right eye but a total glaucomatous optic atrophy was noted. Fundus examination of the left eye showed a near total glaucomatous optic atrophy (vertical disc diameter of 2.1 mm, 0.9:1 cup disc ratio with bipolar notch) as well as foveal hypoplasia. As the IOP was uncontrolled with topical 0.5% timolol maleate eye drops and 0.15% brimonidine tartarate eye drops, the patient underwent trabeculectomy with Mitomycin C under local anesthesia in his left eye. Partial anterior vitrectomy was performed at the same time.

**Figure 2:**
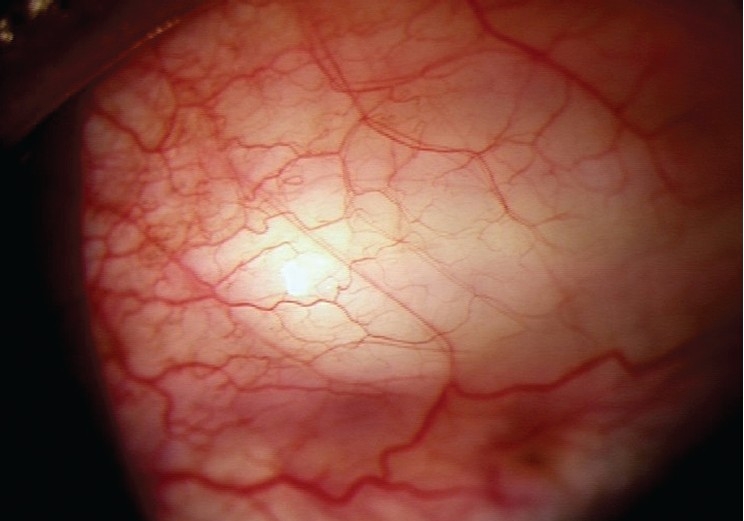
Left eye of the patient with diffuse bleb (postoperatively)

At five weeks postoperatively the patient maintained a best-corrected visual acuity of 20/400 (using + 9 Dsph and 1 diopter cylinder (D cyl) at 180 degree) in the left eye [Fig. 2]. At the three-month follow-up the vision remained the same; there was a diffuse bleb and the IOP was 7 mm Hg without any anti-glaucoma medications. When last seen (six months postoperatively), the best-corrected visual acuity of 20/400 was maintained; the IOP was 6 mm Hg without any anti-glaucoma medications.

## Discussion

The visual function in aniridia varies from near normal to blindness. The more serious cases where blindness occurs are not due to the aniridia but due to associated conditions like cataract, glaucoma, foveal hypoplasia, corneal dystrophy, and nystagmus. Deletion or mutations involving the PAX6 gene have been implicated in the pathogenesis of various anterior segment anomalies including congenital aphakia.[3] However, to our knowledge there is no reported case in literature of aniridia associated with congenital aphakia. Such an association could be expected to occur by a chance alone in approximately one in 490,000,000 live births. (Chance associations calculated based on approximate prevalence of aniridia in one in 70,000 live births and approximate prevalence of congenital aphakia in one in 70,000 live births.)

This case highlights the rare association of aniridia with aphakia and secondary glaucoma.
